# Land use not litter quality is a stronger driver of decomposition in hyperdiverse tropical forest

**DOI:** 10.1002/ece3.3460

**Published:** 2017-10-04

**Authors:** Sabine Both, Dafydd M. O. Elias, Ully H. Kritzler, Nick J. Ostle, David Johnson

**Affiliations:** ^1^ Institute of Biological and Environmental Sciences University of Aberdeen Aberdeen UK; ^2^ Lancaster Environment Centre Lancaster University Lancaster UK; ^3^ Centre for Ecology & Hydrology Lancaster Environment Centre Lancaster UK; ^4^ School of Earth and Environmental Sciences The University of Manchester Manchester UK

**Keywords:** Borneo, leaf litter chemistry, litterbags, old‐growth forest, phospholipid fatty acids, Sabah, selectively logged forest

## Abstract

In hyperdiverse tropical forests, the key drivers of litter decomposition are poorly understood despite its crucial role in facilitating nutrient availability for plants and microbes. Selective logging is a pressing land use with potential for considerable impacts on plant–soil interactions, litter decomposition, and nutrient cycling. Here, in Borneo's tropical rainforests, we test the hypothesis that decomposition is driven by litter quality and that there is a significant “home‐field advantage,” that is positive interaction between local litter quality and land use. We determined mass loss of leaf litter, collected from selectively logged and old‐growth forest, in a fully factorial experimental design, using meshes that either allowed or precluded access by mesofauna. We measured leaf litter chemical composition before and after the experiment. Key soil chemical and biological properties and microclimatic conditions were measured as land‐use descriptors. We found that despite substantial differences in litter quality, the main driver of decomposition was land‐use type. Whilst inclusion of mesofauna accelerated decomposition, their effect was independent of land use and litter quality. Decomposition of all litters was slower in selectively logged forest than in old‐growth forest. However, there was significantly greater loss of nutrients from litter, especially phosphorus, in selectively logged forest. The analyses of several covariates detected minor microclimatic differences between land‐use types but no alterations in soil chemical properties or free‐living microbial composition. These results demonstrate that selective logging can significantly reduce litter decomposition in tropical rainforest with no evidence of a home‐field advantage. We show that loss of key limiting nutrients from litter (P & N) is greater in selectively logged forest. Overall, the findings hint at subtle differences in microclimate overriding litter quality that result in reduced decomposition rates in selectively logged forests and potentially affect biogeochemical nutrient cycling in the long term.

## 1 INTRODUCTION

Microbial and faunal communities associated with soils and litter have crucial roles in regulating numerous ecosystem functions including decomposition, carbon storage, and nutrient cycles. Understanding the myriad factors that regulate the activity of these communities is therefore an essential goal in ecology. One key factor that shapes the activity of decomposer communities is the nature of the litter that they process; chemical and physical properties of litter are known to directly influence the activity of decomposer communities across different ecosystem types (Cornelissen et al., 1999), including tropical forests (e.g., Paudel et al., 2015), and at different geographical scales (e.g., Garcia‐Palacios, Mckie, Handa, Frainer, & Hattenschwiler, 2016). Substantial modifications in leaf litter properties can arise through large‐scale land‐use perturbations that change plant community species composition and diversity. One biome of particular concern is tropical forest which, worldwide, is threatened by deforestation and conversion into agricultural land (Hansen et al., 2013) but has key roles for global carbon storage (Malhi et al., 2009), in regulating climate (Gedney & Valdes, 2000) and as a reservoir of biodiversity (Myers, Mittermeier, Mittermeier, da Fonseca, & Kent, 2000).

Degraded and secondary forests already comprise around 60% of all tropical rainforest (FAO 2010; Laurance, Sayer, & Cassman, 2014). Logging and land clearance for conversion to agriculture are the predominant threats to tropical forest (Sodhi, Koh, Brook, & Ng, 2004; Wilcove, Giam, Edwards, Fisher, & Koh, 2013) with the highest rates of logging occurring in insular Southeast Asia (Edwards, Tobias, Sheil, Meijaard, & Laurance, 2014; Stibig, Achard, Carboni, Rasi, & Miettinen, 2014). Substantial economic opportunities, mainly from oil palm, pulp, and timber plantations, have led to the rapid and large‐scale transformation of rainforests over recent decades (Hansen et al., 2013). Tropical forests across Southeast Asia harbor a comparatively high density of valuable timber trees, mainly belonging to the Dipterocarpaceae family. Because over two‐thirds of canopy trees in Bornean primary forests are dipterocarps (Cannon, Kartawinata, Leighton, & Peart, 1994), these forests have been subject to strong logging pressure (Achard et al., 2002; Berry et al., 2010; Sodhi et al., 2004). The practice of selective logging, where only larger trees above a prescribed diameter threshold are logged, results in vast areas of disturbed forests (Pfeifer et al., 2016). Unlike most other families of tropical trees, dipterocarps form ectomycorrhizal, symbiotic fungal associations. It has been shown that the selective removal of these species can lead to a substantial reduction in the abundance and diversity of ectomycorrhizal fungi (Kerfahi, Tripathi, Lee, Edwards, & Adams, 2014) or compositional changes in ectomycorrhizal fungi communities (McGuire et al., 2015) with potential implications for rates of biogeochemical cycling. Despite removal of trees and the inevitable generation of numerous forest gaps, total tree species richness is surprisingly constant and compensated by a high abundance and density of pioneer species (Cannon, Peart, & Leighton, 1998; Putz et al., 2012; S. Both unpublished data). The resultant shifts in the composition of plant communities lead to changes in ecosystem scale leaf traits and consequently litter quality (Bakker, Carreño‐Rocabado, & Poorter, 2011; Fortunel, Garnier, & Joffre, 2009). Plant communities show a fundamental trade‐off between leaf traits associated with either resource acquisition or resource conservation (Bakker et al., 2011; Poorter & Bongers, 2006). In line with the leaf economic spectrum (Reich, 2014; Wright et al., 2004), it is expected that old‐growth forest will be dominated by shade‐tolerant, well‐protected leaves with a high leaf dry matter content, high fiber content, and high C: N ratio, resulting in slow decomposition. Whereas in selectively logged forest, a high abundance of fast‐growing pioneer species drives the production of leaves and associated traits to allow maximal tree growth, characterized by high nutrient concentration and low fiber content. The association between plant litter attributes and specialized local decomposer communities can result in a “home‐field advantage” (Gholz, Wedin, Smitherman, Harmon, & Parton, 2000; Veen, Freschet, Ordonez, & Wardle, 2015), but the patterns appear to be inconsistent (Ayres et al., 2009). To date, it is unclear how the outcomes are transferrable to highly diverse forest ecosystems modified by selective logging.

In addition to directly modifying key microbial groups and litter traits, the removal of biomass modifies the stand structure and linked abiotic conditions. The creation of forest gaps and logging roads generates fragmentation and edge effects, leading to changes in microclimatic patterns (Hardwick et al., 2015) and key soil properties (Pinard, Barker, & Tay, 2000; Sidle, Sasaki, Otsuki, Noguchi, & Abdul Rahim, 2004). Hence, selective logging leaves behind a heterogeneous mosaic of altered biotic communities and abiotic conditions, with the potential to impact microbial and faunal decomposer activities. For example, Luke, Fayle, Eggleton, Turner, and Davies (2014) showed that selective logging led to a threefold reduction in the abundance of termites in a Southeast Asian forest, and there is evidence that conventionally logged forest in Borneo supported a different community composition to old‐growth forest (Hasegawa et al., 2014). However, there have been no experiments that explicitly test the impact of these changes in mesofauna on litter decomposition in response to logging.

It has been suggested that functional diversity at different trophic levels can be retained in selectively logged forest (Barnes et al., 2014; Konopik, Gray, Grafe, Steffan‐Dewenter, & Fayle, 2014) and that degraded forests remain of surprisingly high ecological value. Given that on a global scale, human‐modified forests dominate over pristine forests (Edwards et al., 2014; Laurance et al., 2014), we urgently need to improve understanding of these land use changes on key ecological processes such as litter decomposition and soil nutrient cycling.

In this study, we aim to unravel the top–down effects of selective logging on leaf decomposition. We quantified litter decomposition rates in two different forest types, old‐growth (OG) and selectively logged (SL) tropical rainforest in Sabah, Malaysian Borneo. In order to disentangle the habitat effect from the leaf litter effect, we conducted a multifactorial litterbag decomposition experiment with reciprocal introduction of leaf litter from OG and SL forest into those two land‐use types. Mass loss of leaf litter was determined after 24 weeks and chemical composition analyzed at the beginning and the end of the experiment. In addition, we quantified key soil chemical and biological properties, including phospholipid fatty acids (PLFA), and microclimatic conditions as a properties of habitat change from old‐growth to selectively logged rainforest. In order to evaluate how macroinvertebrate communities affected decomposition, we used two different mesh sizes to exclude or allow mesofauna access to the leaf litter.

We hypothesized that (1) leaf litter quality would differ between land‐use types and that these differences (2) would translate into altered decomposition rates. We expected that the interplay of abiotic and biotic properties would lead to a (3) “home‐field advantage,” that is positive interaction between land‐use type and locally derived litter quality. Given that logging may lead to significant negative impacts on the abundance of decomposers, we expected that (4) access to litter by macroinvertebrates would have little effect on decomposition in logged forests.

## 2 MATERIAL AND METHODS

### 2.1 Study site, litter, and soil preparation

The study was conducted in Sabah, Malaysian Borneo. We chose the Maliau Basin Conservation Area, centered on 4°49′N, 116°54′E with a size of 588 km^2^, as an example of old‐growth dipterocarp forest, representing the typical vegetation of lowland Borneo (Hazebroek, Adlin, & Sinun, 2004). Tree species diversity in Sabah is high: almost 400 tree species have been recorded in 4 ha of old‐growth forest (in nearby Danum Valley Conservation Area, Newbery, Campbell, Lee, Ridsdale, & Still, 1992).

Additionally, we focussed on selectively logged forest as a human‐modified land‐use type, a dominant habitat in Sabah generated due to the transformation of old‐growth forest into oil palm plantation. Despite the selective removal of trees, species richness remains high (Cannon et al., 1998) and continuously increases with time since disturbance (Slik, Verburg, & Keßler, 2002). Logging occurs on a vast scale in Borneo, and we chose the closest accessible and experimentally tractable area of selectively logged forest, which was located within the Stability of Altered Forest Ecosystems Project (SAFE, Ewers et al. 2011). The area was historically connected to the Maliau Basin Conservation Area but is now fragmented due to logging. The linear distance between the Maliau Basin and the SAFE project site is approximately 50 miles. The climate is moist tropical, with no distinct dry season and an estimated annual precipitation of approximately 3,800 mm (Mykura, 1989). We conducted a full‐factorial litterbag experiment, with the factors land use (two levels: old‐growth forest [OG], selectively logged forest [SL]), litter type (two levels: OG, SL), and mesh size (two levels: fine, coarse). In each land‐use type, we set up eight pairs of study plots, consisting of one plot per litter type each (paired as part of an additional experiment). The two plots forming a pair were directly next to each other. The mean elevation of the plots was 273 m and 454 m a.s.l. in OG and SL, respectively. The location of plots was chosen to capture a representative part of the forest type. However, in order to avoid extreme environmental differences, we avoided the center of big forest gaps. Within land‐use type, pairs were on average 50 m apart. Plots were 2 m × 3 m and protected against litter fall by suspending a coarse fishing net 50 cm above. A total of 16 plot pairs were established in October 2014. Microclimatic variables (air temperature, relative humidity, soil temperature, and soil moisture) were measured hourly over the course of the experiment next to each plot pair (HOBO U23 Pro V2 Temp/RH and Delta‐T SM300 Soil Moisture/Temperature).

Soil samples were collected from each plot pair (*N* = 16) by hammering a 5‐cm diameter plastic pipe into the soil to 15 cm depth. In the laboratory, soil was extracted from the core, the organic horizon measured and separated from the underlying mineral soil, and both fresh sample masses recorded. These were homogenized by sieving to 4 mm and then separated and weighed into subsamples for soil pH determination, PLFA extraction, and soil C, N, and P. Subsamples for soil C, N, and P analysis (with stones and roots) were air‐dried at 25°C for at least 10 days, reweighed, sieved to 2 mm, and the volume of stones and roots measured. A subsample of the air‐dried, sieved soil was oven‐dried at 105°C for 24 hr and mass loss recorded. The remaining soil was dried to 65°C and ball milled (Fritsch, Germany) to a fine, homogeneous powder. Subsamples for PLFA analysis were frozen, freeze‐dried (Christ Alpha 4 Plus, Germany), sieved to 2 mm, and then ground to a fine powder using a pestle and mortar.

In October 2014, freshly fallen leaf litter was raked‐up from representative areas nearby the experimental study plots and sorted to omit visibly, partially decomposed leaves, woody, and reproductive plant material. Leaf litter was cut by hand into pieces of ca. 20 cm^2^ size, oven‐dried at 50°C, and well mixed. While this approach risks introducing litter that has started to senesce, it has the dual advantage of producing uniform litter that more effectively captures characteristics of species‐rich forests. We used the litterbag method to measure litter mass loss (Harmon, Nadelhoffer, & Blair, 1999). Litterbags measuring 15 cm × 15 cm were made of 50 and 2,000 micron nylon mesh (fine and coarse, respectively) to selectively exclude or include different components of the decomposer community. Fine mesh allows access by bacteria, fungal hyphae, and nematodes. Coarse mesh enables access of Collembola, Acari, and other medium‐sized decomposers (mesofauna).

Litterbags with either fine or coarse mesh were filled with 10 g oven‐dry leaf litter mixture of either OG or SL. In November 2014, these litterbags were placed into the experimental plots. Analyses are based on 128 litterbags (2 land‐use types × 2 litter types × 2 mesh sizes × 2 replicates × 8 plot pairs). Litterbags were collected 24 weeks after the start of the experiment. After collection, litterbags were rinsed quickly to remove soil particles. Rinsing was standardized and as short as possible in order to minimize nutrient leaching (usually 60 s). In order to account for contamination by ingrowing roots and hyphae, litterbags were sorted by hand and leaf litter was separated. The content of litterbags was oven‐dried for 72 hr at 50°C and weighed. Decomposition was determined as percent mass loss at the end of the experiment.

### 2.2 Chemical analyses of leaf litter and soil

Chemical composition of initial leaf litter was measured in replicated bulk samples (*N* = 5 per litter type) as follows: total C and N were quantified on an automated elemental analyzer (NCS 2500, CE Instruments, UK); total P was assessed using a sulfuric acid/hydrogen peroxide digest and subsequently measured by flow injection (FIAstar™ 5000, Foss Tecator, Denmark). Base cations Ca, K, and Mg were measured with atomic absorption spectroscopy (AAS, Perkin Elmer AAnalyst 100, MA, USA) after sulfuric acid digestion. Lignin and cellulose were analyzed by sequential digestion of fibers using an ANKOM Fiber Analyzer (ANKOM Technology, Macedon, NY, USA). Elemental analyses were also performed on the residual litter material at the end of the experiment, on a reduced set of samples (one replicate, i.e., *N* = 64). Total soil C and N was measured on a LECO Truspec elemental analyser (LECO Corporation, USA). Total P was measured using a sulfuric acid/hydrogen peroxide digest and subsequently measured using a SEAL AutoAnalyzer 3 (Seal Analytical, UK). Soil pH was determined on fresh subsamples in a 2.5:1 water : soil slurry suspension using a pH probe calibrated between pH 4–7 (pH210 Meter, Hanna Instruments, UK). Bulk density of each horizon was calculated using values of moisture loss from the air and oven‐dried subsamples following methods in the GB Countryside Survey (Emmett et al., 2008; Reynolds et al., 2013).

### 2.3 Soil microbial community

Soil microbial PLFAs were extracted as part of the total lipid extract from subsamples of freeze‐dried, ground organic soil, using a chloroform‐methanol extraction modified from White, Davis, Nickels, King, & Bobbie (1979). Identification of PLFAs was carried out on a gas chromatograph (Agilent Technologies 6890) fitted with a CP‐Sil 5CB fused—silica capillary column (50 m × 0.32 mm i.d. × 0.25 μm) and a flame ionization detector. Sample PLFA peaks were identified based on known relative retention times calculated as a proportion of the internal standards (C13—methyl tridecanoate and C19—methyl nonadecanoate [Avanti Polar Lipids, Inc.]). The terminal and mid‐chain branched fatty acids C15:0i, C15:0a, C16:0i C17:0i, and C17:0a were used as indicators of gram‐positive bacteria (Whitaker et al., 2014). Cyclopropyl saturated and monounsaturated fatty acids 16:1ω7c, 7,8 cyclic C17:0, C18:1ω7c, and 7,8 cy‐C19:0 were used as indicators of gram‐negative bacteria (Rinnan & Baath, 2009). The fatty acids C18:2ω6,9c and C18:1ω9c were taken as indicators of fungi (Kaiser, Frank, Wild, Koranda, & Richter, 2010). Total microbial biomass was taken as the total of all identified PLFAs (C14:0, C15:0, C16:1, C16:1ω5c, C16:0, C17:1ω8c, 7Me—C17:0, br17:0, br18:0, C18:1ω5c, C18:0, C19:1 plus those listed above).

### 2.4 Data analysis

Differences in initial leaf litter quality based on litter origin were analyzed with ANOVA. Soil properties, PLFA data, and microclimate of study plots were analyzed with linear mixed effect models with the fixed factor land‐use type and plot nested within land‐use type as random factor.

To evaluate the effects of the experimental treatments on litter decomposition, we developed a linear mixed‐effects model with mass loss (% mass loss of dry weight compared to *t* = 0) as the response variable. We checked for potential outliers with the Tukey's method which uses an interquartile range approach. Three outliers were detected; however, they had a very minor contribution to the overall data set. Taking out the outliers decreased the average mass loss by 0.44%. Due to this small effect and the potential ecological implications, we decided against outlier removal. The response variable was normally distributed as confirmed by Shapiro–Wilk test. Land‐use type (OG, SL), litter type (OG, SL), and mesh size (fine, coarse) and their interactions were treated as fixed factors. Plot nested within plot pair was included as a random factor.

To detect which environmental covariates measured might generate the impact of land‐use type, we decomposed the factor “land use” into the variables that were found to be significantly different between forests and most likely to impact litter mass loss. In order to reflect the experimental design, we forced the selection of the fixed factors litter type and mesh size. We performed a model selection comparing the values of Akaike's information criterion (AIC), which is an index of the goodness of fit and indicates how well the model agrees with the data.

A principal component analysis (PCA) was performed on the chemical composition of initial litter types and the residual litter at the end of the experiment. To test the changes in leaf litter quality, we calculated Δ concentration for each chemical parameter and performed the same linear mixed‐effects model with Δ concentration as response variable and fixed factors land‐use type, litter type, mesh size and plot nested within plot pair as random factor.

All analyses were performed with R 3.3.2 (R Core Team, 2016), using the packages MuMIn and vegan.

## 3 RESULTS

### 3.1 Initial leaf litter and environmental properties

Chemical analyses of the two initial litter types showed various significant differences in composition (Table 1). Litter from old‐growth forest had almost 50% higher concentration of phosphorus (*p* = .001) and a much lower C:P ratio (*p* = .002). The content of C was similar in both litter types, but litter from selectively logged forest had a significant higher C:N ratio (*p* = .036) and N:P ratio (*p* = .013). Calcium content was 20% higher in OG litter (*p* = .02). With regard to fiber concentration, OG litter had significantly higher soluble cell content (*p* < .0001) and consequently lower nonsoluble cell content (*p* = .001). SL litter had a significantly higher concentration of cellulose as well as lignin and other recalcitrant components (*p* = .002 and *p* = .003, respectively).

**Table 1:** Chemical properties of leaf litter collected in the two differing land‐use types and used in the decomposition experiment, shown are mean values and their standard deviation. Significant differences derived from ANOVA are indicated with ***p < .001, **p < .01, *p < .05. Five subsamples were analyzed for each litter type

Chemical properties	Old‐growth forest	Selectively logged forest
Total N %	1.54 ± 0.09	1.38 ± 0.19
Total C %	45.8 ± 0.69	47.6 ± 2.61
P mg/g	0.84 ± 0.08	0.59** ± 0.08
C:N	29.9 ± 2.04	34.9* ± 3.97
C:P	550.9 ± 54.14	812.1** ± 115.26
N:P	18.5 ± 1.87	23.3* ± 2.84
Cations		
Ca mg/g	17.5 ± 1.59	14.8* ± 1.33
Mg mg/g	1.95 ± 0.26	2.30 ± 0.22
K mg/g	4.92 ± 0.68	5.68 ± 0.71
Fibers		
Soluble cell content %	55.7 ± 2.74	46.4*** ± 2.81
Nonsoluble cell content %	40.1 ± 2.89	49.1** ± 2.71
Cellulose %	19.5 ± 1.15	22.1** ± 0.51
Lignin and recalcitrant %	13.6 ± 1.60	19.1** ± 2.41

Chemical properties emphasize a higher quality of leaf litter in OG forest compared to SL litter, reflected in high P and N concentration, low C:N and C:P ratio, and lower concentration of fibers.

There was no significant difference in soil properties of the two land‐use types, neither for organic nor mineral soil horizon. Only the C:N ratio in the mineral soil horizon of selectively logged forest was significantly higher compared to OG mineral soil (13.3 ± 2.5 (mean value ± standard deviation) and 10.9 ± 1.6, respectively, *p* = .038, Table 2).

**Table 2:** Characteristics of soil from all experimental study plot pairs (*N* = 16; mean and standard deviation). Significant differences between old‐growth and selectively logged forest within each horizon were derived from a linear mixed model and are indicated with *p < .05

Soil variables	Old‐growth forest		Selectively logged forest	
	O‐Horizon	M‐Horizon	O‐Horizon	M‐Horizon
Bulk density	0.71 ± 0.17	1.2 ± 0.2	0.76 ± 0.23	1.23 ± 0.3
pH	5.33 ± 0.5	5.27 ± 0.17	4.9 ± 0.55	4.96 ± 0.42
Total C %	4.33 ± 1.54	1.28 ± 0.52	5.6 ± 2.87	1.4 ± 0.53
Total N %	0.28 ± 0.08	0.12 ± 0.05	0.29 ± 0.12	0.11 ± 0.03
Total P (μg/g)	306.9 ± 91.77	197.1 ± 89.46	269.3 ± 64.93	147.2 ± 46.05
C:N	15.1 ± 3.14	10.9 ± 1.62*	18.2 ± 3.28	13.3 ± 2.45*
C:P	145.2 ± 52.32	69.7 ± 26.53	200.6 ± 76.44	96.5 ± 30.55

**Figure 1:**
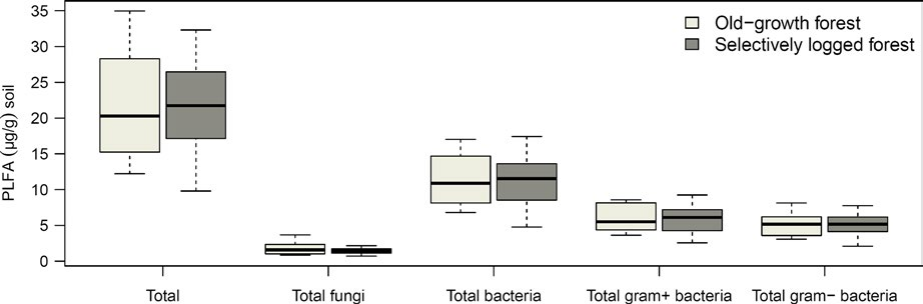
Phospholipid fatty acids (PLFA) concentrations in soil from experimental study plots (*N* = 16). Data presented in μg PLFA g^−1^ soil dry weight and shown separately for OG and SL forest plots. No significant differences were detected with mixed effect models of PLFA concentration and land‐use type. Figure shows median, upper and lower quartiles, and 95% confidence intervals

Analyses of PLFA indicated no significant differences between total PLFA concentration, bacterial (gram‐positive [G_pos_] and gram‐negative [G_neg_]), and fungal PLFA between OG and SL (Figure 1). Ratios of G_pos_:G_neg_ bacteria and fungal PLFA:Bacterial PLFA was also not significantly different between land uses.

Continuous microclimate measurements revealed several significant differences between the two land‐use types (Table 3). OG forest had significantly higher mean temperature (*p* = .045), higher maximum temperature (*p* = .005), and lower minimum temperature (*p* = .008) compared to SL forest. However, despite being significant, the absolute temperature differences were very small, for example only 0.3°C for mean temperature. Mean air humidity was also significantly different (*p* < .0001) between land‐use type, but the differences were also small (97.7% ± 0.9 in OG plots compared to 92.6% ± 2.3 in SL plots).

**Table 3:** Microclimatic measurements across the experimental study plots of old‐growth forest (OG) and selectively logged forest (SL). Significant differences derived from ANOVA are indicated with ***p < .001, **p < .01, *p < .05

Forest type	Air temperature (°C)			Relative humidity (%)			Soil temperature (°C)			Soil moisture (%)		
	Mean	Max	Min	Mean	Max	Min	Mean	Max	Min	Mean	Max	Min
OG	24.2* ± 0.19	30.2** ± 0.31	19.4** ± 0.65	97.7*** ± 0.90	100 ± 0	66.0 ± 2.98	24.8** ± 0.33	26.2 ± 0.78	23.4** ± 0.35	32.9 ± 2.67	49.3 ± 3.25	18.8 ± 3.09
SL	23.9* ± 0.29	29.5** ± 0.43	20.3** ± 0.53	92.6*** ± 2.25	100 ± 0	62.9 ± 3.86	24.1** ± 0.35	27.4 ± 4.06	22.7** ± 0.31	30.6 ± 6.59	56.3 ± 18.92	17.6 ± 7.14

**Figure A1:**
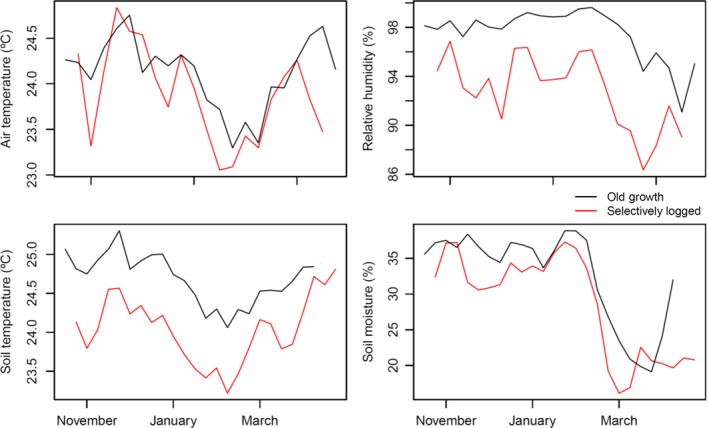
Mean microclimate variables over the course of the experiment (November 2015–April 2016). All data from all eight plots per site were averaged over a timespan of a week, and the course of weekly mean values is displayed

Soil mean temperature was significantly higher in OG plots (*p* = .001) but again the difference was only minor, with 24.8°C ± 0.3 and 24.1°C ± 0.4 in OG versus SL plots. The minimum soil temperature was also greater in OG compared to SL plots with 23.4°C ± 0.4 also significantly higher in OG compared to SL with 22.7°C ± 0.3 (*p* = .001). Weekly averaged values show that microclimatic differences were consistent over the course of the experiment (Figure A1).

### 3.2 Mass loss and litter quality

**Figure 2:**
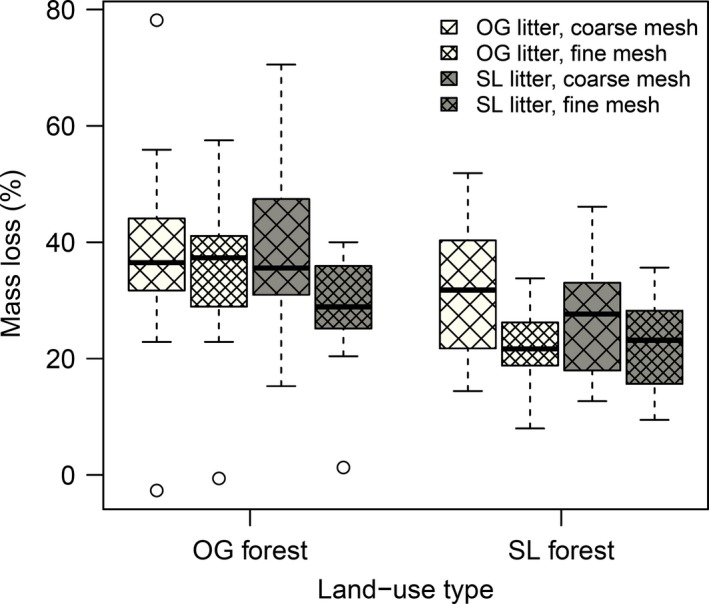
Mass loss in percent per treatment at the end of the decomposition experiment. Shown are the three experimental factors: land use, litter type, and mesh size. Figure shows median, upper and lower quartiles, 95% confidence intervals, and outliers (>1.5 times of quartile), OG = old‐growth, SL = selectively logged, OG litter white, SL litter gray boxplots with mesh size indicated by cross hatch. Statistical analysis presented in Table 4

After 24 weeks of incubation, the mass loss ranged from 0% to 78% across the experiment, the mean mass loss was 30.3% across all treatments (Figure 2). Despite this remarkable range, the variance was not related to experimental treatments.

At the end of the experiment (*N* = 128), results of the linear mixed‐effects model revealed significant effects of land‐use type with 36% mass loss in OG forest (*p* = .003, Table 4). There was no effect of litter type on mass loss and no interaction between litter type and land use. Mesh size significantly affected mass loss with fine mesh across all treatments decreasing mass loss by 24% (*p* = 0.001, Table 4).

**Table 4:** Results of the mixed‐effects models for treatment effects on litter mass loss at the end of the experiment (*N* = 128). Numerator degrees of freedom (DFn), denominator degrees of freedom (DFd), *F*‐values, and *p*‐values are shown. Significant values in bold, **p < .01

Fixed effects	DFn	DFd	*F*	*p*
Land use (LU)	1	14	13.177	.003**
Litter type (LT)	1	14	1.95	.184
Mesh size (MS)	1	92	11.523	.001**
LU × LT	1	14	0.002	.963
LU × MS	1	92	0.035	.851
LT × MS	1	92	0.015	.904
LU × LT × MS	1	92	2.396	.125

Model selection revealed that when replacing land‐use type with the covariates which were found to be significantly different between land uses, the best model selected for minimum and maximum temperature, mineral soil C:N ratio, and the interactions between mineral soil C:N ratio with litter quality and mesh size. We allowed for both variables of temperature measures in the model because they may reflect different processes occurring during decomposition (Table A1). Soil moisture, soil temperature, and air humidity did not increase the quality of the model and did not significantly affect mass loss in our study system.

**Figure 3:**
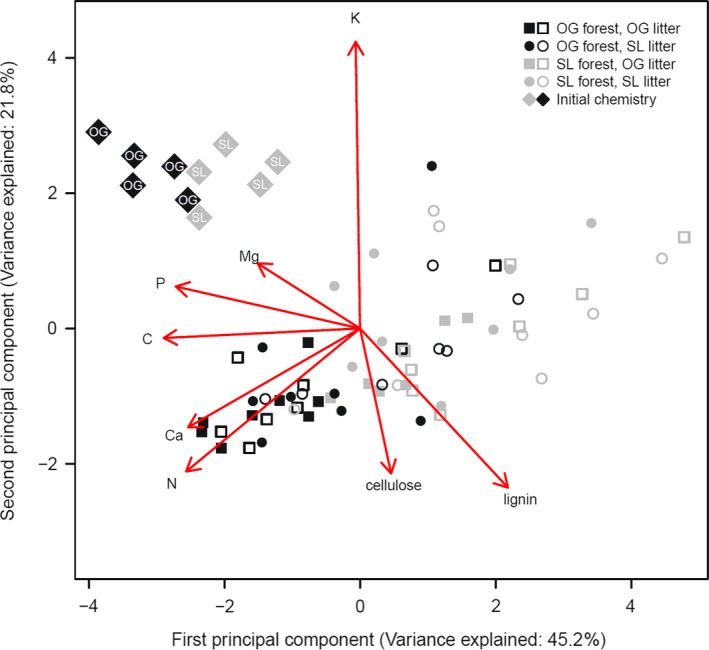
Principal component analysis biplot of leaf litter quality of initial litter mixtures (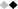) and residual litter bag content. Factor land‐use type is given by color with old‐growth forest (OG) in black, selectively logged forest (SL) in gray. OG litter type (■□) and SL litter type (●○). Mesh size is indicated by closed (fine) and open (coarse) symbols. Vectors are pointing in the direction of the chemical component, the angle, and relative length of vectors to each other reflect their correlations

Principal component analysis showed clear differentiation between initial litter types as well as a distinct change of litter quality due to decomposition (Figure 3). The PCA's first two axes cumulatively explained 67% of the variance. In comparison with the initial litter composition, cellulose and lignin accumulated at the end of the experiment, reflecting the recalcitrant nature of these substances. To some extent, chemical composition in remaining litter clustered according to experimental treatments. Residual litter incubating in OG forest type was characterized by higher concentrations of nitrogen, calcium, and phosphorus and was unaffected by litter type. This result is unexpected given the higher mass loss in OG forest, although the concentration of nutrients was higher compared to SL forest also at the end of the experiment. Potassium concentration was independent from other nutrients (as indicated by the perpendicular position of the arrow in Figure 3).

**Figure 4:**
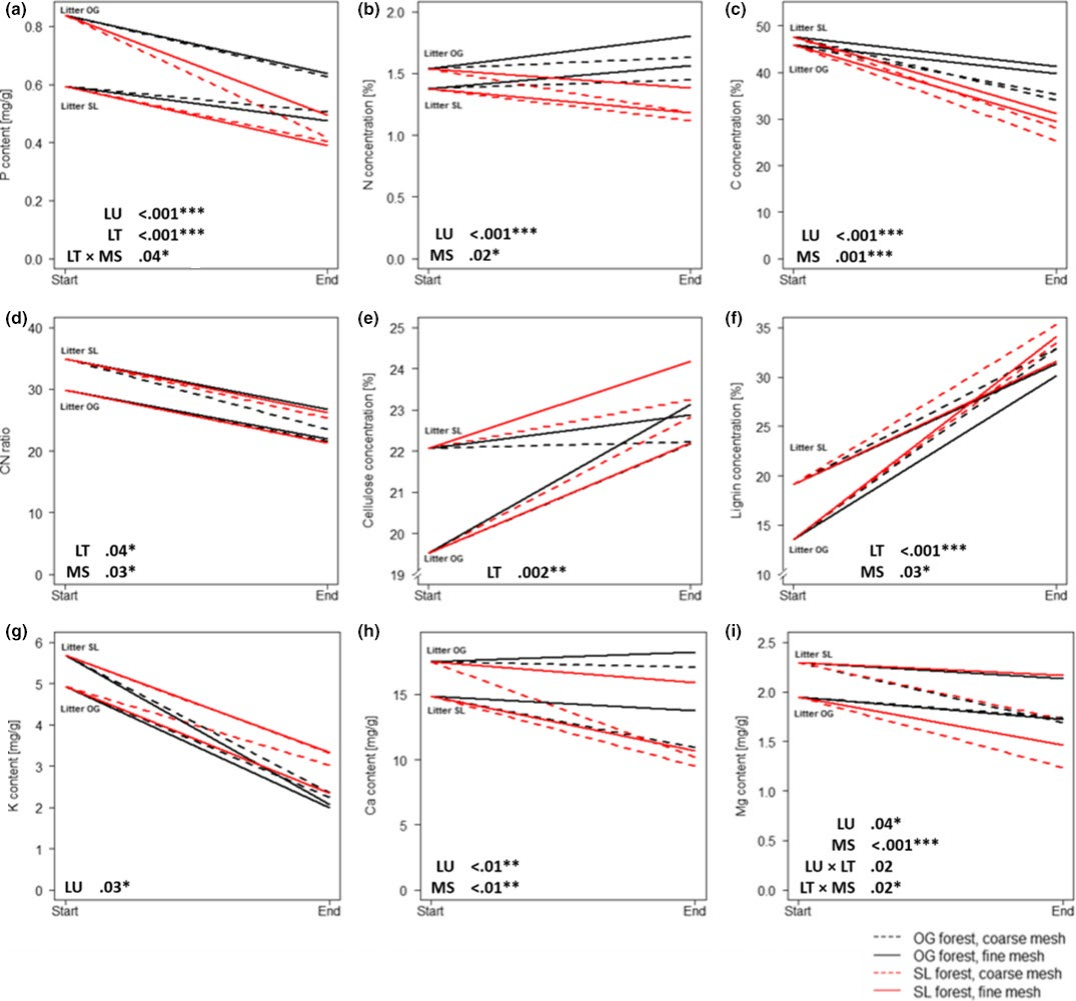
Concentration of chemical variables (a ‐ i) from the beginning of the experiment (“Start”) and at the end of the experiment after 24 weeks (“End”). Land‐use types are shown by color (old‐growth forest: black, selectively logged forest: red), coarse mesh litterbags are shown with dashed lines, and fine mesh litterbags have a solid line. The two litter types are indicated at the start of the lines (OG = old‐growth forest litter, SL = selectively logged forest litter). Significant factors and *p*‐values are shown and indicated with ***p < .001, **p < .01, *p < .05. Results are based on mixed‐effects models with land use (LU), litter type (LT), and mesh size (MS) as fixed factors and plots as random factor. Response variable is the change of nutrient concentration (in %) at the end of the experiment (*N* = 64)

The patterns and magnitude of responses of elemental concentrations varied among experimental treatments (Figure 4). The change of P concentration was significantly affected by land use and litter type (both *p* < .0001, Figure 4a). There was an interaction between litter type and mesh size with SL litter having lowest P concentration at the end of the experiment in fine mesh bags (*p* = .04, Figure 4a). Figure 4a shows that despite starting with a higher P concentration, the OG litter decomposing in SL forest had the same P concentration as SL litter at the end of the experiment. When decomposing in SL forest, P concentration was 32% lower across all treatments compared to OG forest. By contrast, the change of N and C concentration was significantly affected by land‐use type and by mesh size but not by litter type (Figure 4b,c). Cellulose and lignin concentrations increased over the course of the experiment, independent of litter type, reflecting the preferential decomposition of soluble compounds and accumulation of fibers due to their lower decomposition capacity. Several interacting effects were detected namely between litter type and mesh size for P, K, and Mg concentration (Figure 4a,g,i).

## 4 DISCUSSION

We report one of the first studies that simultaneously test the effect of leaf litter quality and land use on leaf litter decomposition in tropical forest. Our study shows that a major driver of litter decomposition was land‐use change and that substantial differences in litter quality associated with selectively logged and old‐growth forest had no significant effect. We also discovered faster decomposition rates in undisturbed forest compared to selectively logged forest, which supports both our first hypothesis and previous work in a similar system (Yeong, Reynolds, & Hill, 2016). In general, decomposition was slow compared to other studies in tropical forests (Hättenschwiler, Coq, Barantal, & Handa, 2011; Powers et al., 2009) but comparable to mass loss reported by Mayor and Henkel (2006). When considering abiotic factors, we found that temperature as well as the C : N ratio of mineral soil had the greatest influence on litter decomposition with higher mean and maximum air temperature in old‐growth forest plots driving faster rates of decomposition. This may reflect changes to the stand structure as a result of logging although differences in the elevation between forest types may also be a factor. Because the microclimatic differences were much smaller than it would be expected from the difference in elevation, we assume that the variations detected are consequences from the land‐use change (Hardwick et al., 2015).

Although we confirmed our hypothesis that the initial leaf litter quality would vary significantly between the two land‐use types, the differences were the opposite of what we expected. We predicted that SL forest litter would have higher N and lower fiber content due to fast‐growing pioneer species in line with the leaf economic spectrum (Wright et al., 2004) and thus, greater decomposability. This was not the case, and instead, leaf litter of the OG forest had higher nutrient content which was especially pronounced for P. Soil properties were comparable between forest types and therefore do not explain this effects on litter quality. Other work has shown that the immense richness of both plant species and leaf traits in tropical rainforests results in leaf litter quality that is highly variable and locally clustered (Scherer‐Lorenzen, Bonilla, & Potvin, 2007; Townsend, Asner, & Cleveland, 2008; Townsend, Cleveland, Asner, & Bustamante, 2007), and so it is challenging to capture the underlying patterns in litter collections. Both of the forest types studied here have high tree species richness. Permanent 1 ha plots in near proximity to the experimental plots host more than 130 tree species in OG forest and more than 200 tree species in SL forest (individuals exceeding 10 cm diameter at breast height). The number of trees per ha is surprisingly similar, but SL forest is characterized by forest gaps and smaller tree diameters (S. Both, unpublished data). Disentangling the relevance of this heterogeneity in hyperdiverse forests should be a focus of future experiments.

We could not support our second hypothesis that the leaf litter with higher quality would decompose faster. On the contrary, despite having strikingly higher nutrient quality, the OG litter did not decompose faster in SL forest, which we would have expected if litter quality is the main driver of decomposition. This finding is in contrast to other studies (e.g., Cornwell et al., 2008; Dale, Turner, & Bardgett, 2015) where differences in chemical composition translated into modified decomposition. Our results are in line with Lohbeck, Poorter, Martínez‐Ramos, and Bongers (2015) who also showed that community‐level plant characteristics were of minor relevance for in situ decomposition compared to overall stand characteristics.

The results are surprising given that the tree communities at each location generated leaf litter that differed significantly in a number of key traits (notably P, C:N, C:P, N:P ratios and fiber content). When focussing on the changes in element concentration and the impact of experimental treatments, interesting and ecologically important effects emerged: Despite higher mass loss in OG forest, there was a higher loss of key elements in SL forest, which could be attributed to selective removal by plants, immobilization by soil biota or leaching. Even with slower decomposition, there was significantly greater loss of selected nutrients, particularly for P, in SL forest. The initial nutrient composition and loss of P from litter might indicate that plants and their associated microorganisms have adapted to greater P limitation in SL forest. Because our analyses did not detect land‐use differences in amounts and proportions of bacterial and fungal PLFA, the pattern of selective loss of nutrients from litter might hint at changes in the functional microbial composition. Decomposition is accelerated by bacterial and fungal exoenzymes as well as the nutrient demand and competition among microbes, and these complex processes could have been modified by microbial species turnover without species loss (Craine, Morrow, & Fierer, 2007; Mooshammer et al., 2012; Waring, 2012).

Our results confirm the absence of a home‐field advantage as it also had been observed in Brazilian rainforest (Gießelmann et al., 2011). In order to detect such an effect, we would expect a characteristic soil and microbial signature in the respective forest type or a strong specialization of the decomposer community. In spite of noticeable tree removal due to selective logging and a modified tree composition resulting in abundant pioneer species (S. Both, unpublished data), our study revealed that the soil chemical and microbial properties were remarkably similar in both land‐use types. The soil microbial communities under tropical forests can be resilient to disturbance from logging (Lee‐Cruz, Edwards, Tripathi, & Adams, 2013; Tripathi et al., 2016), which may apply to our study system. However, despite similar free‐living microbial community composition, differences in the rate of loss of nutrients from litter suggest functional differentiation between communities.

Albeit only small but significant differences were observed, microclimate appeared to be more suitable for faster decomposition in OG forest, while SL forest plots showed a higher variance of microclimatic conditions. The SL forest stand structure is reflected in these microclimatic variables, where canopy gaps and edge effects mediate heterogeneous abiotic conditions. OG forest appears to be able to buffer microclimatic extremes and hence allows consistently suitable decomposition conditions. In our study, both forest types lie in the same geographic area emphasizing the relevance of small‐scale microclimatic differences modified by selective logging (Hardwick et al., 2015).

Our results also highlight the importance of the invertebrate community, reflected in a strong effect of mesh size on decomposition. Litterbags with coarse mesh excluded macrofauna, whereas fine mesh inhibited access to mesofauna. In the absence of mesofauna, decomposition was around 24% less compared to litterbags allowing their access. The impact of these decomposers was comparable between land‐use types, given that the mesh effect showed no interaction with land use, rejecting our 4th hypothesis. Our results indicate that the functioning of the mesofauna community is resilient to the intensity of perturbation imposed by selective logging, at least with regard to decomposition. Our data does not allow conclusions regarding the impact of macrofauna which is expected to be important too, based on studies showing a significant reduction in the abundance of key termite taxa (Luke et al., 2014) and detritivorous fauna (Woodcock et al., 2013) in Malaysian Borneo. It is possible that loss of faunal decomposition may have been compensated by other organisms, such as mycorrhizal fungi that can act as regulators of decomposition (Hättenschwiler et al., 2011; Read & Perez‐Moreno, 2003). One of the specific features of Southeast Asian rainforests is the dominance of the dipterocarp family, which are colonized exclusively by ECM fungi (Brearley, 2012), and their selective logging likely drives local extinction of these symbionts and changes in fungal community composition (Kerfahi et al., 2014). One potential consequence of a reduction in the abundance of ECM fungi is to change the competitive dynamics with saprotrophic fungi, where the removal of ECM fungi allows saprotrophic fungi to increase in activity and accelerate decomposition (Gadgil & Gadgil, 1971). Our results suggest this effect was not seen and support more recent manipulative experiments of ECM fungal abundance in South American rainforest (Mayor & Henkel, 2006). One possible explanation is that AM fungi associated with nondipterocarps fulfilled a similar niche to ECM fungi and prevented saprotrophs from increasing their competitiveness.

A shift in the dominant mycorrhizal group as a result of selectively logging may also have contributed to the contrasting patterns of P loss from the litter. It is possible that the AM fungal dominated community at the selectively logged site actively acquired and immobilized leaf litter P with greater efficiency. Phosphorus limitation is thought to be the key selective pressure driving evolution of AM fungi and, at least in temperate systems, these fungi are typically adept at acquiring P (Smith & Read, 2008). More studies are needed to shed light on the interplay of ECM and AM fungi and their role in decomposition and P partitioning, particularly in human‐modified tropical landscapes (Turner, 2008).

Generally, responses of taxonomic groups to disturbance are complex and play in concert with the ecosystem functions they contribute to (Newbold et al., 2014), and therefore, multitrophic approaches need to complement our current knowledge. We cannot precisely define the driver of enhanced decomposition in OG forests, but our data suggest that the composition of abiotic and possibly biotic factors has a greater effect on decomposition than in SL forests. Despite a strong effect of land use, the forest sites studied did not differ in basic soil properties, microbial communities (sensu Hasegawa et al., 2014), and the activity of mesofauna. The impact of modest differences in microclimate had a strong effect on decomposition, as has been shown in other studies (Cusack, Chou, Yang, Harmon, & Silver, 2009; Dale et al., 2015). The initial striking difference in leaf litter quality was compensated by the habitat properties. Our results emphasize that realized decomposition plays in concert with a variety of factors such as microclimate, soil properties, and the decomposer community (Lohbeck et al., 2015). In our study system, microclimatic properties exerted a stronger influence on decomposition than litter quality. Although we analyzed several environmental variables, they proved to be of limited explanatory power. Disentangling causations within human‐modified, species‐rich ecosystems requires ongoing research, particularly with regard to the consequences for ecosystem functioning (Bakker et al., 2011; Lohbeck et al., 2015). The higher litter quality of OG forest as well as higher decomposition rates in this land‐use type emphasize the importance of old‐growth forests for provision of valuable ecosystem services. Our results do not support the assumption that degraded forests retain similar ecosystem functioning relative to OG forests (Berry et al., 2010; Gibson et al., 2011), at least not for decomposition and nutrient cycling. Instead, the study adds to the growing evidence that ecosystem processes in tropical forests are sensitive to even small changes in abiotic conditions, and loss of key plant and associated microbial taxa as a result of land‐use pressure.

## CONFLICT OF INTEREST

None declared.

## AUTHOR CONTRIBUTIONS

SB, DJ, DE, and NO designed the study. SB, DE, and UK collected the data; SB, DE, and UK contributed to the chemical sample analyses, SB analyzed the data and led the writing of the manuscript and all authors contributed critically to the drafts and gave final approval for publication. All authors declare no conflict of interest.

## Appendix 1

### 1.1

**Table A1:** Results of the mixed‐effect model with the best fit based on AIC after decomposing the factor land‐use type into environmental variables which were found to differ between forests. Numerator degrees of freedom (DFn), denominator degrees of freedom (DFd), *F*‐values, and *p*‐values are shown. Significant values in bold, **p* < .05, ***p* < .01

Fixed effects	DFn	DFd	*F*	*p*
Litter type (LT)	1	14	1.985	0.180
Mesh size (MS)	1	94	11.736	**0.001****
Maximum temperature	1	12	9.968	**0.008****
Minimum temperature	1	12	8.533	**0.013***
Mineral soil C:N	1	12	9.147	**0.011***
Mineral soil C:N × LT	1	14	2.285	0.153
Mineral soil C:N × MS	1	94	2.112	0.150
